# FOXO3 on the Road to Longevity: Lessons From SNPs and Chromatin Hubs

**DOI:** 10.1016/j.csbj.2019.06.011

**Published:** 2019-06-13

**Authors:** Paola Sanese, Giovanna Forte, Vittoria Disciglio, Valentina Grossi, Cristiano Simone

**Affiliations:** a Medical Genetics, Department of Biomedical Sciences and Human Oncology (DIMO), University of Bari Aldo Moro, Piazza G. Cesare, 11, 70124 Bari, Italy; b Medical Genetics, National Institute of Gastroenterology ‘S. de Bellis’ Research Hospital, Via Turi, 27, 70013 Castellana Grotte (BA), Italy

**Keywords:** *FOXO3*, Longevity, Aging, SNP, Chromatin hub, IGF-1, Insulin growth factor-1, FOXO3, Forkhead box 3, FHRE, Forkhead response element, ROS, Reactive oxygen species, SOD2, Superoxide dismutase 2, SNV, Single nucleotide variant, LD, Linkage disequilibrium, SNP, Single nucleotide polymorphism, GWAS, Genome-wide association study, HPS, Hamartomatous polyposis syndrome, PJS, Peutz-Jeghers syndrome, PHTS, PTEN hamartoma tumor syndrome, 3C, Chromosome conformation capture, ACH, Active chromatin hub, TAD, Topologically associated domain, HSE, Heat shock element, 5′UTR, Five prime untranslated region, HSF1, Heat shock factor 1, GPx, Glutathione peroxidase, ER, Estrogen receptor, ERE, Estrogen-responsive element

## Abstract

Health span is driven by a precise interplay between genes and the environment. Cell response to environmental cues is mediated by signaling cascades and genetic variants that affect gene expression by regulating chromatin plasticity. Indeed, they can promote the interaction of promoters with regulatory elements by forming active chromatin hubs.

*FOXO3* encodes a transcription factor with a strong impact on aging and age-related phenotypes, as it regulates stress response, therefore affecting lifespan. A significant association has been shown between human longevity and several *FOXO3* variants located in intron 2. This haplotype block forms a putative aging chromatin hub in which *FOXO3* has a central role, as it modulates the physical connection and activity of neighboring genes involved in age-related processes.

Here we describe the role of *FOXO3* and its single-nucleotide polymorphisms (SNPs) in healthy aging, with a focus on the enhancer region encompassing the SNP *rs2802292*, which upregulates FOXO3 expression and can promote the activity of the aging hub in response to different stress stimuli. *FOXO3* protective effect on lifespan may be due to the accessibility of this region to transcription factors promoting its expression. This could in part explain the differences in *FOXO3* association with longevity between genders, as its activity in females may be modulated by estrogens through estrogen receptor response elements located in the *rs2802292*-encompassing region. Altogether, the molecular mechanisms described here may help establish whether the *rs2802292* SNP can be taken advantage of in predictive medicine and define the potential of targeting FOXO3 for age-related diseases.

## Graphical Abstract

**Figure:**
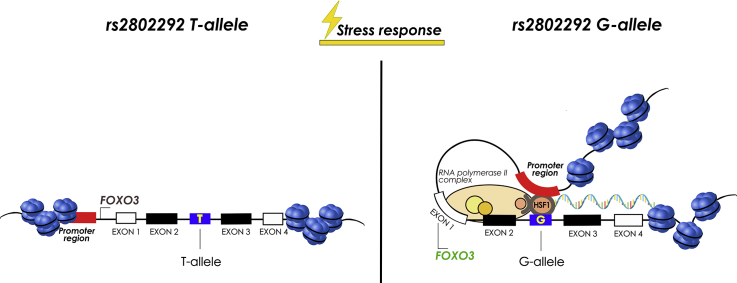

## 1 Introduction

Healthy aging is the result of a fine regulation of the biological processes that occur in response to environmental and physiological signals. These processes support lifespan extension and are mediated by nutrient and stress sensors. Under stress conditions, the expression and activity of sensors and genes are modulated in a way that promotes cell protection and maintenance, and eventually extends lifespan. Indeed, loss of cell homeostasis is a common event in age-related diseases and cancer. An intricate network of genetic factors and signaling pathways have been identified as coordinators of cell response to physiological or environmental changes [[1], [2], [3], [4]]. These mechanisms often involve hormones (e.g. estrogens), which control growth and development, and are responsible for maintaining healthy and normal functions of tissues and organs, ultimately affecting aging processes [5].

The contribution of genetic factors to healthy aging is significant [6]. Many genetic variants have been associated with increased risk of age-related diseases, and genetic polymorphisms or mutations that affect the functions of major sensors and effectors have been linked to longevity, both in model organisms and in humans. Studies on longevity are usually carried out in populations of centenarians, whose lifespan is nearly twice the predicted mean of the relevant population at the time of birth [7]. Aging is also governed by signaling pathways typically involving coordinated cascades of modifications, which in turn alter protein conformation, activity, stability, interaction, and subcellular localization. In the classical view, these pathways begin at the cell surface and extend into the nucleus, where they affect the interactions of transcription factors with the chromatin template. The ultimate endpoint of signal transduction is often considered to be a modification of chromatin architecture modulating DNA accessibility and gene expression. However, such complex biological features cannot be explained by a “one-way street” ending at chromatin; instead, they imply a model in which different hubs direct multilayered and multidirectional regulatory networks.

In this framework, nine hallmarks of aging have been identified. These include genomic instability, telomere attrition, epigenetic alterations, loss of proteostasis, deregulated nutrient sensing, mitochondrial dysfunction, cellular senescence, stem cell exhaustion, and altered intercellular communication. These hallmarks contribute in varying degrees to the aging process and collectively determine the aging phenotype [4].

One of the major pathways involved in the regulation of the aging rate is the insulin/IGF-1 cascade, which negatively influences the activity of *FOXO3*, one of the most prominent genes associated with human longevity [8]. This transcription factor is a key regulator of aging-related processes, acting as a hub for molecular signaling and chromatin conformation.

In this review, we will focus on the importance of *FOXO3* in human aging and longevity by discussing findings related to its genetic variants, cis-regulatory elements that modulate its activity in lifespan, and its “central” role in an aging hub. We will also provide a full picture of *FOXO3* by dissecting its network of functions involved in the aging processes, with the final goal of ascertaining whether it can be taken advantage of for prognosis and therapy response in age-related diseases, and/or used as a target to improve human health during aging.

## 2 FOXO3 Activity Sustains Longevity

The forkhead box (FOX) is a heterogenic protein family that includes >100 transcription factors, which can be divided into 19 subclasses based on phylogenetic analyses. Its name refers to a conserved DNA-binding domain, a sequence of 80 to 100 amino acids called the forkhead domain, which is shared by Fox proteins [9]. Fox transcription factors are involved in a plethora of diverse functions, including developmental regulation, metabolism, and tumorigenesis. Their function is tightly regulated through interaction with various binding partners, such as co-activators, co-repressors and other transcription factors [10].

FOXO proteins have been identified in several species, including nematodes (*Caenorhabditis elegans*)*,* zebrafish (*Dario rerio*), fruit flies (*Drosophila melanogaster*), mice, and humans [11]. The four members of the FOXO class, i.e. FOXO1, FOXO3, FOXO4, and FOXO6, share high sequence homology and have different activities and subcellular localizations. To exert their role as transcription factors, FOXO proteins bind through their DNA binding domain to the consensus motif 5′-TTGTTTAC-3′ (the forkhead response element, FHRE) within the promoter sequence of their target genes [12]. Then, their C-terminal transactivation domain initiates gene transcription, acting as a transcriptional activator or repressor based on the specific range of recruited co-factors. Most FOXO proteins are ubiquitous but are not equally expressed in tissues and organs. As such, they can regulate different genes depending on the cell type, thereby showing functional specificity [13]. Interestingly, the first identified FOXO member, DAF-16, which regulates larva formation in *C. elegans*, has been proven to increase lifespan and regulate nutrient sensing by mediating insulin-like metabolic signaling, as well as resistance to stress [[14], [15], [16], [17]]. In fact, under environmental conditions, mutations in DAF-16 lead to defective larva formation [18]. Moreover, AKT-mediated phosphorylation of DAF-16 negatively regulates longevity in response to insulin/IGF-1 [8]. This function seems to be evolutionarily conserved. Indeed, dFOXO, the *Drosophila melanogaster* FOXO homologue, affects insulin signaling and extends lifespan when expressed in the adult fat body [19,20]. In mice, *Foxo3*-null females show abnormal ovarian follicular development leading to degeneration and age-dependent infertility (around 12 weeks) [21,22]. Interestingly, *Foxo3* knock-out animals also exhibited a decreased rate of glucose uptake in glucose tolerance tests after an overnight fast [22].

FOXO3 is an evolutionarily conserved transcription factor regulating the expression of genes involved in several biological processes [23]. In particular, its expression is associated with age-related phenotypes in multiple tissues [24]. Several studies have been conducted to elucidate the mechanisms by which FOXO3 influences longevity. We now know that FOXO3 is a master regulator and sustains lifespan in response to various stimuli, including hormones, growth factors, and nutrients, which promote specific FOXO3-mediated gene expression programs regulating stress resistance, metabolism, cell cycle arrest, and apoptosis [24]. The first and most studied pathway reported to modulate FOXO3 activity in longevity processes is the insulin/IGF-1/PI3K signaling cascade, whose impairment is associated with extended lifespan in a variety of organisms including yeasts, worms, flies and mice [25].

Notably, it has been shown that FOXO3 has a pivotal role in oxidative stress response, DNA damage, starvation, and caloric restriction with the final effect of increasing lifespan [12]. Specifically, FOXO3 protects cells from reactive oxygen species (ROS) accumulation through the regulation of genes involved in cell detoxification and survival [26,27]. In fact, it is upregulated in response to ROS accumulation, which leads to increased expression of its downstream transcriptional targets manganese superoxide dismutase 2 (SOD2) [26] and catalase [27], two enzymes with a key role in ROS detoxification. Furthermore, FOXO3 is essential for protecting cells from DNA damage, since it regulates the expression of proteins involved in DNA conservation and repair, such as GADD45A [28]. Caloric restriction induces metabolic changes that positively influence tissue-specific effectors of longevity pathways, leading to a reduced aging rate. FOXO3 has been found to be involved in the protective effect of dietary restriction, thereby modulating aging [29]. Recently, a mitochondrial fraction of FOXO3 has been uncovered [[30], [31], [32]]: in mammalian myotubes and fibroblasts, AMPK-mediated FOXO3 accumulation has been observed in mitochondria upon glucose restriction [30]. This leads to the formation of a transcriptional complex containing FOXO3, SIRT3 and the mitochondrial RNA polymerase at mitochondrial DNA regulatory regions, thereby promoting expression of the mitochondrial genome and a subsequent increase in oxygen consumption. The AMPK-FOXO3 axis is also required in the modulation of the balance between oxidative phosphorylation and glycolysis in response to metabolic stress [30,33]. These observations suggest a role for FOXO3 in lifespan extension in an AMPK-dependent manner.

Based on its multiple functions, FOXO3 acts as a mediator of biological processes that promote lifespan and prevent aging-related diseases [29]. Alterations of these processes are involved in cardiovascular diseases, type 2 diabetes [12,34], neurodegenerative diseases, and cancer [[35], [36], [37]]. Indeed, FOXO3 has a role in the regulation of genes involved in autophagy: in response to decreased glycolysis in colorectal and ovarian cancer cells, it first induces autophagy as an attempt to retain energy to survive, whereas it triggers autophagic cell death under persistent stress conditions [[38], [39], [40]]. FOXO3 was one of the first FOXO factors recognized as a tumor suppressor in human breast cancer since its absence correlated with poor patient survival [41]. Moreover, low levels of FOXO3 protein expression have been associated with poor prognosis in several types of tumors, including ovarian cancer, hepatocellular carcinoma, gastric cancer, and lung adenocarcinoma [[42], [43], [44], [45], [46], [47]]. Its inhibition promotes cell transformation, tumor progression, and angiogenesis, while its overexpression inhibits breast tumor [41,48,49]. Furthermore, it has been shown that ERK- and AMPK-mediated phosphorylation of FOXO3 in response to metabolic stress leads to its cleavage and translocation into the mitochondria, where it binds to mitochondrial DNA and activates the expression of mitochondrial genes, thereby sustaining the active role of mitochondria in metabolically stressed cancer cells [33]. In old mice, decreased expression of Foxo3 negatively influences anti-inflammatory responses and its knockout induces premature aging of the enteric nervous system [50]. Additionally, a role for FOXO3 has been suggested in patients with Duchenne muscular dystrophy [51], while increased FOXO3 phosphorylation was observed in chronic obstructive pulmonary disease patients with sarcopenia [52]. FOXO3 also regulates the fine balance of anti-aging biological processes in muscle, such as the maintenance of skeletal muscle stem cell pools [53] and the regulation of protein turnover [54].

**Fig. 1:**
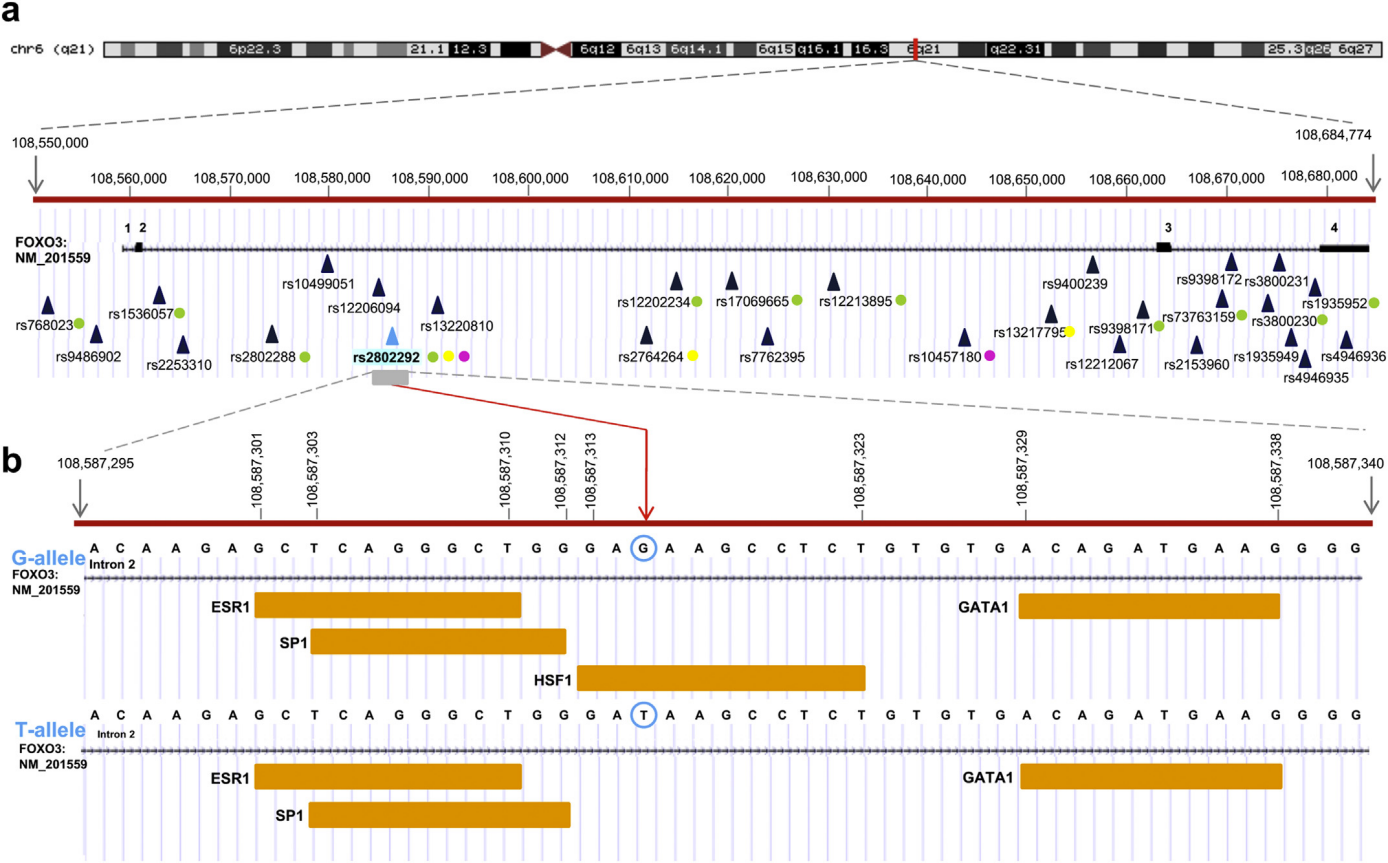
Genomic region encompassing *FOXO3* longevity SNPs *(a) FOXO3* Ref Seq canonical transcript is mapped on the genomic region of chromosome 6 (UCSC Genome Browser, GRCh38). Single-nucleotide polymorphisms (SNPs) associated with longevity are mapped according to the genomic coordinates of the human reference genome and are marked with filled blue triangles. The *rs2802292* SNP is highlighted and marked with a filled blue-sky triangle. Linkage disequilibrium (LD) correlation between *rs2802292* and other SNPs is indicated by filled circles: green circles, LD between *rs2802292, rs2802288, rs768023, rs2253310, rs2802288, rs12202234, rs17069665, rs12212067, rs9398171, rs3800230 and rs1935952*; yellow circles, LD between *rs2802292*, *rs2764264*, and *rs13217795*; pink circles, LD between *rs2802292* and *rs10457180*. (b) chr6:108,587,295-108,587,340. *rs2802292* genomic location is marked with a vertical red arrow, and a blue-sky circle indicates the G/T alternative alleles. Transcription factor response elements (for ESR1, SP1, HSF1, and GATA1), previously identified by in silico analysis [91], are shown as filled orange rectangles. The *rs2802292* G-allele creates a unique binding site for HSF1, while the T-allele fails to do so. The start and the end nucleotide positions for each transcription factor response element are indicated according to the genomic coordinates of the UCSC Genome Browser (GRCh38).

In addition to its role as a transcription factor and modulator of signaling pathways, several studies have shown that *FOXO3* has specific variants that are related to aging. Human *FOXO3* is located on the long arm of chromosome 6 (6q21) and comprises 4 exons and 3 introns, with the two coding exons - exon 2 and 3 – being particularly large (1.4 kb and 4.9 kb, respectively). A human FOXO3 pseudogene, called FOXO3B (later renamed ZNF286B) and comprising only FOXO3 exons 2–4, has been identified on chromosome 17 [55]. Besides the detection of a new p53 binding site in intron 2, which positively regulates FOXO3 expression after DNA damage [56], several genetic association studies have identified *FOXO3* intron 2 as a large sequence of 101,625 bp encompassing various single-nucleotide variants (SNVs) involved in longevity, including *rs2802292*, *rs2802288*, *rs13217795*, *rs2764264*, and *rs2253310*. In particular, *rs2802292* was found in a strong linkage disequilibrium (LD), which is a non-random association of alleles at two or more loci in a given population, with nine out of thirteen putative functional SNPs analyzed by Donlon and colleagues (*rs768023*, *rs2253310*, *rs2802288*, *rs12202234*, *rs17069665*, *rs12212067*, *rs9398171*, *rs3800230*, *rs1935952*), forming a longevity haplotype (Fig. 1a) that is more common in the Asian than in the Caucasian and African populations [57].

## 3 Lessons from *FOXO3* SNPs

SNPs are a variation of the genetic material of a single nucleotide, such that the polymorphic allele is present in the population in a proportion higher than 1%. SNPs cover 90% of all human genetic variations and their density is approximately one SNP every 100–300 bp throughout the entire genome [58]. They can occur within a coding or an intronic region of a gene, or in an intergenic region. SNPs that are not in a coding sequence may compromise splicing or the binding of transcription factors, and they are usually studied to characterize variants and their frequency associated with a disease or other phenotype.

The discovery of an association between a gene variant and a trait subsequently requires the identification of the specific variant that is responsible for the biologic change underlying the observed phenotype, since there could be other closely linked variants in strong LD. In recent years, the relationship between *FOXO3* variants and human longevity has been explored. Numerous SNPs located in intronic regions of the gene have been shown to be associated with longevity with statistical significance in different human populations (Fig. 1a). Intriguingly, all these variants are in a haplotype block on chromosome 6, which has a high degree of LD [57]. Indeed, a 100 kb region in *FOXO3* intron 2 was found to be rich in longevity-associated SNPs. In general, SNPs can affect gene regulation through several mechanisms. For example, they can act as a splice-site variant, as a transcription enhancer element, by inducing an amino acid change, or by influencing chromatin structure [59].

A genetic variation within *FOXO3* was first reported to be associated with human longevity in a study of an American cohort of Japanese ancestry with a mean age of 97.9 years [35]. The authors found that the *rs2764264*, *rs13217795*, and *rs2802292* SNPs had a high LD and were associated with healthy aging. Long-lived men had homozygosity for the G-allele of *FOXO3 rs2802292* and showed lower prevalence of cancer and cardiovascular diseases along with greater insulin sensitivity compared to younger controls. The association of these polymorphisms was validated in Southern Italian Centenarian males [60] and then confirmed in the German and French population, also in women [61]. In the Han Chinese population, *rs2802292* and the novel *rs2253310* and *rs4946936* SNPs were found to be associated with longevity in both genders [62]. In a Danish cohort of oldest-old and middle-aged individuals, eight *FOXO3* SNPs out of fifteen were found to be associated with longevity. Notably, four of them had not been previously reported (*rs12206094*, *rs13220810*, *rs7762395*, and *rs9486902*) [63]. Subsequent studies supported the relationship between *FOXO3* and longevity and identified new variants involved, as is the case for *rs1935949* and *rs4946935* in a Caucasian cohort [64]. A meta-analysis of eleven independent studies with cases and controls from different ethnic groups was carried out to verify the association of eight FOXO3 polymorphisms with longevity. This study confirmed the association for *rs2802292*, *rs2764264*, *rs13217795*, *rs1935949*, and *rs2802288*, and identified the effects of *rs2802292* and *rs2764264* as male-specific [65]. Another meta-analysis that included genome-wide association study (GWAS) data from the CHARGE consortium revealed that *rs10457180* was in LD with *rs2802292* and supported the existence of a statistically significant association with longevity [66]. Another meta-analysis of eight case-control studies showed that the *rs2802288* A-allele and *rs2802292* G-allele were beneficial to longevity in a subgroup of Southern Chinese individuals [67].

All these studies strongly corroborated the association between *FOXO3* genetic variants and longevity; however, the underlying mechanisms remain to be elucidated. There is evidence showing that some *FOXO3* SNP alleles may be protective against age-related diseases. For example, in Japanese women an association has been described between longevity-associated *FOXO3* variants and lower blood pressure and essential hypertension [68]; it was also found that *FOXO3 rs2153960* G-allele is associated with lower circulating IGF-1 levels, which are protective against insulin resistance-related diseases and mortality [69].

The first study on *FOXO3* genetic variants implicated in longevity revealed that homozygosity for the *rs2802292* G-allele is associated with a lower prevalence of coronary artery disease and cancer [35]. Subsequent studies confirmed the importance of this variant in longevity and age-related diseases. In a population of oldest-old Danes, minor alleles of *FOXO3* variants, including the *rs2802292* G-allele, were reported to be associated with higher activity of daily living and fewer bone fractures [37]. *rs2802292*, together with *rs10457180*, was also associated with cardiovascular disease incidence in a recent study on older Swedes [36]. A cohort study of older American men confirmed the association between *FOXO3* variants and cause-specific mortality for major causes of death, including coronary heart disease, cancer, and stroke; specifically, the *FOXO3 rs2802292* G-allele was associated with a 10% reduction in all-cause mortality. In older Japanese, White and Black American men, it was found that the longevity-associated *rs2802292* G-allele was a protective factor for coronary artery disease mortality [70].

Despite the correlation detected between *FOXO3* SNPs and age-related diseases, the molecular mechanisms by which the protective alleles reduce mortality and promote human longevity are not fully understood. Two intronic *FOXO3* SNPs have been associated with increased FOXO3 expression and greater transactivation activity [55]. In a recent study on patients with hamartomatous polyposis syndromes (HPS), an inverse correlation between the *rs2802292* G-allele and cancer risk has been described. These syndromes are characterized by mutations in *STK11* (Peutz-Jeghers syndrome, PJS) or *PTEN* (PTEN hamartoma tumor syndrome, PHTS) [71], which encode for FOXO3 upstream regulators [29]. Indeed, longevity-associated SNPs that do not act as protein-coding variants may reside in regulatory regions responsible for the activation of gene expression [72]. For example, it was recently shown that the intronic *rs2802292* G-allele is associated with increased FOXO3 basal expression [71,73] and that homozygosity for the G-allele correlates with lower frequency of age-related diseases in centenarians [35]. These data suggest that *FOXO3* intron 2, and in particular the *rs2802292* SNP, may act as a regulatory region. Confirmation of this hypothesis may be important to unveil the functional role of this polymorphism and could deepen our understanding of *FOXO3* role in human longevity and healthy aging.

*rs2802292* is located at genomic position 108,587,315 of chromosome 6 (human reference genome GRCh38), and the global population frequency of the alternative alleles G and T is 0.53 and 0.47, respectively (1000 Genome Project Phase III). The frequency of the *rs2802292* G-allele varies among the different 1000 Genome Project super populations, i.e. Africans (G-allele frequency: 0.83), Americans (0.39), Europeans (0.43), East Asians (0.31), and South Asians (0.56), thus it represents the minor allele in Western populations (Americans, Europeans).

## 4 Active Chromatin Hub: *rs2802292* Plays Enhancer Functions

Chromatin organization and spatial conformation are known to have a key role in the regulation of gene expression programs [[74], [75], [76]]. Gene transcription is regulated by cis-regulatory elements, which are sequences located in regions of non-coding DNA both in close proximity and in loci distal from the coding region, often tens or hundreds of kilobases away and with unrelated genes in between. All cis-regulatory elements (promoters, enhancers, insulators, etc.) operate as three-dimensional structures, which come into contact by means of specific spatial conformations via chromatin looping [[77], [78], [79]]. This was first documented thanks to the development of a new technique called chromosome conformation capture (3C) [80], by which mouse beta-globin genes have been demonstrated to spatially interact with promoters and upstream regulatory elements through the formation of an active chromatin hub (ACH) [81,82]. The transcriptional outcome of an ACH is strongly influenced by the spatial interaction of cis-regulatory elements. Additional cis-regulatory elements at the edge of these domains (i.e. insulators) may stabilize the interactions in the ACH and maintain the required expression levels [83]. Thus, chromosomes are organized in chromatin globules through the interaction of specific regions, called topologically associated domains (TADs) [84], which are evolutionarily conserved [85] and cell type-specific [86]. This suggests that these TADs have functional implications, such as the regulation of the expression of genes included in each domain, and that they are required to establish lineage-specific expression programs defining cellular identity [87]. CTCF was among the first proteins reported to mediate chromatin looping between its binding sites [88].

Many non-coding genetic variants, including SNPs, within risk-associated loci alter gene expression in a disease- and tissue-specific manner by modulating the activity of cis-regulatory elements, especially enhancers, through mechanisms involving transcription factor binding. For example, breast cancer risk-associated SNPs modulate chromatin affinity for FOXA1 and alter TOX3 expression [89]. Moreover, genetic variants can alter chromatin loop formation by bridging enhancers and promoters, as is the case for the SNP located in the *OCA2* gene enhancer, which promotes chromatin looping and is associated with darker pigmentation in melanocytes [90].

Recently, it was shown that the 90 bp sequence around the *FOXO3* intronic SNP *rs2802292* has enhancer functions and that different types of cellular stresses induce the recruitment of HSF1, which promotes FOXO3 expression [91]. HSF1 is an evolutionarily highly conserved transcription factor that plays a role in stress-induced transcription. Besides its main activity in regulating the expression of chaperone heat shock proteins that protect cells from cellular insults, HSF1 is involved in different physiological processes, including development, metabolism, and aging, and controls various loci participating in stress response [92]. Prolonged lifespan has been described as being dependent on both HSF1 and FOXO, which are both negatively regulated by insulin/IGF-1 signaling.

**Fig. 2:**
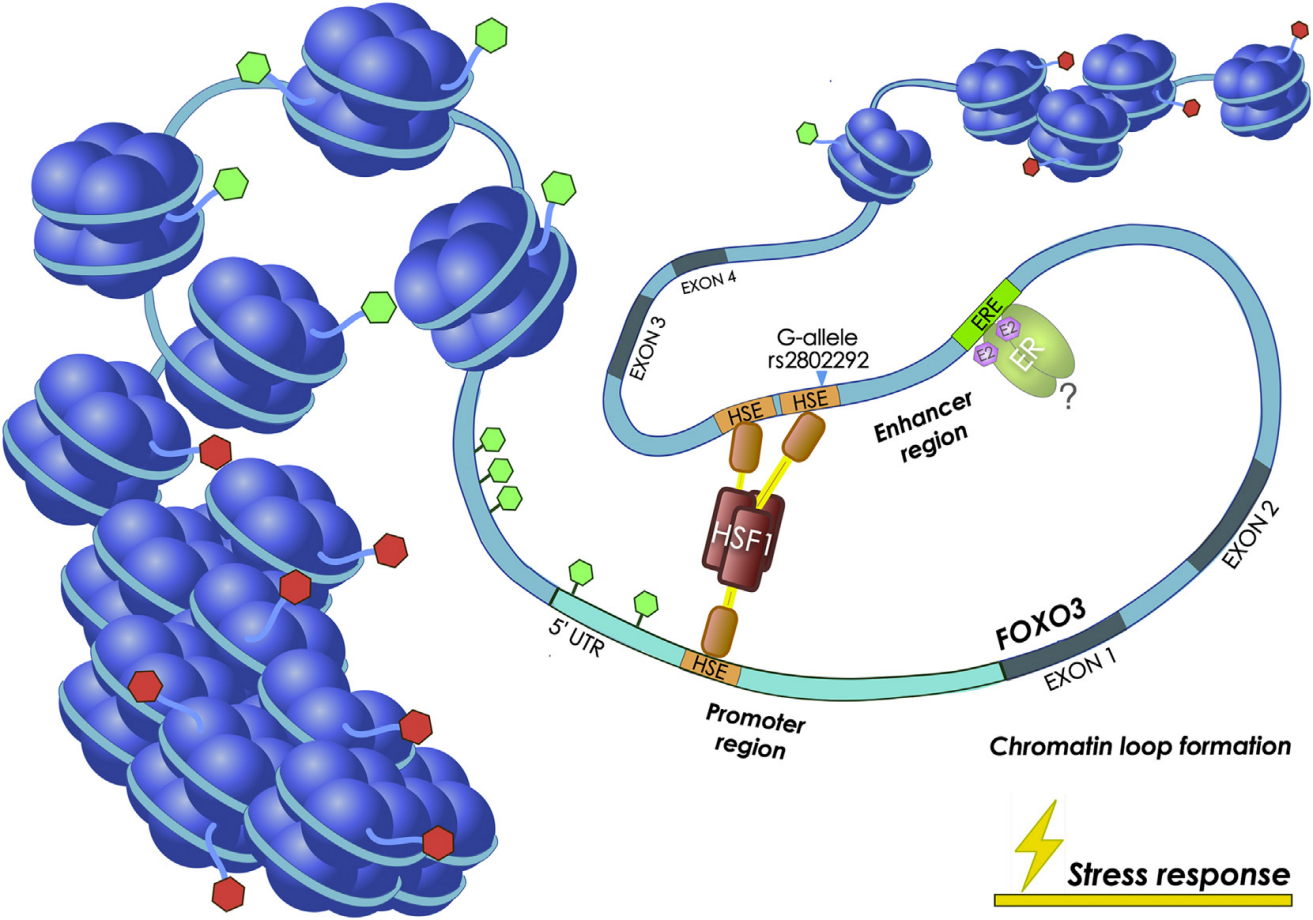
Enhancer role of the region encompassing *rs2802292* in response to stress stimuli. The *rs2802292* locus has enhancer functions. *FOXO3* effect on lifespan extension may be dependent on the accessibility of this region to transcription factors that positively regulate FOXO3 transcription. The presence of the G-allele creates a HSF1 binding site, which induces promoter-enhancer interaction by chromatin looping, thereby fostering FOXO3 expression and the activity of the aging hub. In female individuals, the association with longevity may be dependent on estrogen-mediated ER activity in this region. Green and red flags on chromatin (nucleosomes and DNA filament) indicate active and inactive epigenetic marks, respectively, which regulate the accessibility of regulatory and binding elements to transcription factors. HSF1: heat shock factor 1; ER: estrogen receptor; E2: estradiol; HSE: heat shock element; ERE: estrogen responsive element; 5’UTR: five prime untranslated region. (For interpretation of the references to colour in this figure legend, the reader is referred to the web version of this article.)

The above-mentioned study revealed that a unique heat shock element (HSE) DNA binding site for HSF1 is created by the presence of a G at *FOXO3* intronic SNP *rs2802292* (Fig. 1b). HSF1 binds as a homotrimer to its cognate HSE regulatory site, composed of a minimum of two inverted repeats of the 5-bp consensus sequence 5′-nGAAn-3′. The loop domain of HSF1 dictates DNA-binding specificity and response to heat stress [93]. Consistently, an extended analysis of the sequence around the *rs2802292* SNP revealed an organization corresponding to a stepped (STP) type HSE [94]. After the first nGAAn sequence, there is a potential third pentameric unit, nTTCn, that should further stabilize HSF1 binding. Alternatively, the third pentameric unit for HSF1 binding could be the HSE found in the 5′UTR [91]. Of note, it has been previously shown by M. Fernandes and colleagues that a G > T change in the second position of the HSE pentameric unit (nGAAn) reduces HSF1 binding to DNA by >16-fold [95]. This is the case of the T/G-allele at the *rs2802292* locus, where the G-allele is recognized and bound by HSF1, while the T-allele fails to do so. This work revealed that this SNP-encompassing region has enhancer properties and its activation triggers FOXO3 upregulation. At the molecular level, HSF1 mediates the occurrence of a promoter-enhancer interaction at *FOXO3* locus involving the 5′UTR and the *rs2802292* region by chromatin looping (Fig. 2). These data suggest the existence of an HSF1-FOXO3 axis in human cells that could be involved in stress response pathways functionally regulating lifespan and disease susceptibility. Under nutrient, genotoxic, and oxidative stress, homozygosity for the *rs2802292* G-allele increases FOXO3 expression through HSF1 and activates its antioxidant, metabolic, and DNA repair transcriptional programs both in the nucleus and in the mitochondria, leading to increased tolerance to stress [91] (Fig. 2).

These findings revealed the link between *FOXO3* SNPs and cis-regulatory elements, and are in accordance with a recent work showing that 13 SNPs significantly modify the binding of 18 transcription factors involved in growth, differentiation, stem cell maintenance, energy sensing, and muscle homeostasis. These variants were found to be physically linked to elements located in *FOXO3* introns 2 and 3 via RNA polymerase II binding sites and to be clustered together, thus forming a cis-regulatory unit that acts as a longevity haplotype on chromosome 6 [57]. In addition, other work suggested that the A-allele of *rs4946935* creates a binding site for the transcription factor SRF [55].

Uncovering the mechanistic processes underlying the role of the intronic *rs2802292* G-allele is expected to increase our understanding of the molecular mechanisms involved in healthy aging and reduced susceptibility to age-related diseases.

## 5 A *FOXO3* Aging Network Hub

Several studies suggest that chromatin looping forms functional units comprising thousands of base pairs and that an association exists between the tridimensional chromatin organization of a genomic locus and the activity of the genes located in that region. As an example, Hox genes encode proteins that are extremely important for embryonic development and are located in four specific genomic clusters modulating their activity by forming chromatin loops and therefore dynamically moving closer or farther away seven distant regulatory elements. This loop organization modulates the position and thereby guides the expression of genes that have a central role in this chromatin hub [96]. Another study showed that in erythroid cells *TAL1* locus forms a loop hub that facilitates communication between genes and their cis-regulatory elements [97].

Human aging genes tend to be organized in network hubs together with clusters of development genes, suggesting that human aging influences developmental processes [98]. Moreover, it was found that human disease genes are located close to aging genes and that aging genes are strongly associated with essential genes. Thus, it seems that aging networks are in constant communication with diverse functional pathways [99].

Donlon et al. carried out an analysis in order to search for chromatin contact points in *FOXO3* near neighborhood revealing that its promoter is physically connected by chromatin looping to 46 genes which have roles in various cell resilience processes, such as nutrient sensing, cellular stress and damage response, cell cycle, and autophagy [57]. In the proposed model, the pro-longevity haplotype influences the aggregation of transcription factors with the *FOXO3* promoter, and this event promotes *FOXO3* migration toward the transcriptional apparatus and neighboring genes. Together, these elements form a larger functional unit of 7,268,123 bp on chromosome 6, with *FOXO3* being in the center of this extended region. Moreover, the authors found that *FOXO3* migration toward its neighboring genes is greater and its expression is higher in the presence of the G-allele of SNP *rs2802292* compared to TT genotype cell lines. As there were no substantial differences in the expression of neighboring genes, they speculated that the longevity SNPs would promote the activation of FOXO3 expression, thereby indirectly influencing the tissue-specific expression of other genes. These findings suggest that *FOXO3* interactome within a highly conserved region on chromosome 6 acts as an aging chromatin hub and comes into direct contact with cis-regulatory elements and possibly other genes related to aging.

## 6 May *FOXO3* Be Involved in Estrogen Impact on Women Health Span?

Females live longer than males in many mammalian species, including humans, suggesting that there are conserved differences attributed to specific biological characteristics of both genders. The rate of H_2_O_2_ production is crucial in determining lifespan [100], and mitochondria from females have been found to generate approximately half the amount of H_2_O_2_ detected in males [101]. This is due to estrogens, which bind to estrogen receptors (ER) and work by activating MAPK and NF-κB signaling pathways [102,103] and by increasing the expression of longevity-associated genes, including those encoding for the antioxidant enzymes SOD2 and glutathione peroxidase (GPx) [104].

Based on an in silico analysis, the sequence encompassing the *FOXO3* SNP *rs2802292* comprises transcription factor response elements for SP1, GATA1 and ER (ESR1) (Fig. 1b) [91]. Among these, the transcription factor ER is of particular interest, since previous studies have shown its functional connection with FOXO3: in breast cancer cells, ER is involved in the control of tumor aggressiveness through modulation of FOXO3 activity [105], while FOXO3 mRNA and protein were found to be upregulated by estradiol [106]; in prostate epithelial cells, a direct relationship between ERβ and FOXO3 expression has been described, resulting in cell differentiation and maintenance of cells in a quiescent state [107]. Thus, the predicted estrogen-responsive element (ERE) site around *rs2802292* suggests a role for ER in FOXO3 transcriptional regulation and may explain how this SNP influences FOXO3 expression and activity in tissues that are under estrogen control.

Several meta-analyses conducted on *FOXO3* intron 2 SNPs that are associated with longevity and age-related diseases revealed that this association is stronger in males [35,60,63,72]. In particular, analyses conducted on HPS patients, which are at higher risk for cancer during lifetime, revealed a protective effect of *rs2802292* G-allele on males and females both in PHTS and in PJS. However, according to a subgroup analysis carried out for each syndrome, the beneficial effect of the G-allele on cancer risk occurs mainly in HPS males. In particular, the proportion of PJS and PHTS males with cancer carrying an *rs2802292* TT genotype was three times and seven times higher, respectively, than in PJS and PHTS males having at least one G-allele, while lower association effects were observed in female subjects [71]. The stronger association detected in male was hypothesized to be due to the fact that in men the protective effect is mostly dependent on the *rs2802292* G-allele which, under stress conditions, promotes HSF1 recruitment to this region and higher FOXO3 expression [91], while in women it may be mainly due to estrogen-mediated ER activity on FOXO3 regulatory region, thus being less dependent on the presence of the *rs2802292* G-allele (Fig. 2).

*In vivo* studies revealed that Foxo3 is a guardian of the ovarian follicle pool, as *Foxo3*-null mice survive but exhibit premature ovarian failure due to global follicular activation that leads to depletion of functional ovarian follicles and oocyte death, which in women is accompanied by premature aging and infertility [22]. Furthermore, transgenic female mice overexpressing constitutively active Foxo3 showed increased fertility and ovaries with a younger-looking profile, which suggests that Foxo3 negatively regulates oocyte growth and follicular development, and promotes the maintenance of ovarian reserves [108,109].

Thus, considering that *FOXO3* plays a major role in the aging hub uncovered on chromosome 6 [57] and in regulating functional ovarian follicles [22,108,109], together with the fact that progesterone and estradiol negatively regulate primordial follicle activation [110], it might be speculated that the regulation of FOXO3 function in follicle activation involves ER activity on *FOXO3* intronic regulatory regions, as previously proposed (Fig. 2).

Based on the described evidence, one of the mechanisms by which FOXO3 affects aging in female individuals may depend on the decreased levels of estrogens in menopause, which would affect ER-mediated FOXO3 expression thereby contributing to premature aging and infertility. This putative *in vivo* mechanism is consistent with the aging hub model proposed by Donlon and colleagues, where the *FOXO3* region encompassing the *rs2802292* SNP influences chromatin conformation and the activation of neighboring genes [57].

## 7 Summary and Outlook

Studies on the association between longevity and polymorphisms have significant clinical importance. Indeed, *FOXO3* genetic variants have been associated with decreased risk of age-related diseases and longevity. Specifically, *FOXO3 rs2802292* G-allele has been shown to have protective effects on several age-related diseases, as it has been associated with lower prevalence of coronary artery disease and cancer, fewer bone fractures, lower cardiovascular disease incidence, and better self-rated health, which strongly predicts mortality [[35], [36], [37],^70^]. In this light, the *rs2802292* SNP might potentially help predict the risk for oxidative stress- and nutrient-related diseases and cancer, with the presence of a G-allele playing a positive role in limiting these diseases and TT carriers possibly having a higher risk.

On the other hand, the identification of compounds and molecules modulating FOXO3 activity and expression might help improve the effects of conventional therapies for age-related diseases. A healthy aging phenotype has been described in association with energy-restricted diets [[111], [112], [113]] and dietary supplement usage [114]. In this context, the identification of new molecules able to target FOXO3 activity may help develop novel therapeutic approaches for disorders related to cellular metabolism and stress response. Besides curcumin, green tea and other compounds showing beneficial effects on FOXO3 activity [115,116], the anti-diabetic drug metformin and the polyphenol plant chemical resveratrol have been identified as calorie restriction mimetics that trigger FOXO3 expression by activating AMPK. Their ability to mimic calorie restriction might allow to extend lifespan and reduce the risk of age-related chronic pathologies. Notably, it has been found that metformin induces FOXO3 phosphorylation on serine 30 via AMPK activation and affects its nuclear vs. mitochondrial localization and transcriptional activity, thereby sustaining healthy metabolism and potentially benefiting longevity [30,33].

Taken together, the findings outlined in this review support the hypothesis that *FOXO3* is the master regulator of an aging hub. Moreover, they stress the importance of further investigating the *FOXO3* SNP *rs2802292* since it might help identify responders vs non-responders to conventional therapies, calorie restriction and/or dietary supplements, as well as predict the outcome of personalized approaches for age-related diseases. Lastly, the mechanistic explanation proposed for FOXO3 cellular function may foster new directions for future research, since it shows that understanding how SNPs modulate biological processes can shed light on gene function and associated phenotypes.

## Funding

This study was partially supported by a “GIOVANI RICERCATORI GRANT 2011-2012” GR-2011-02351968 from the Italian MOH (to C.S.) and an “Investigator Grant 2014” from the Italian Association for Cancer Research (AIRC) (grant number: IG 15696) (to C.S.).

## Author Contribution

Writing-review & editing, P.S., G.F., V.D., V.G.; supervision, C.S.

## Declarations of Competing Interest

No potential conflicts of interest were disclosed.

## Acknowledgements

We thank Dr. Francesco Paolo Jori for his helpful discussion during the preparation of the manuscript and editorial assistance.
